# A ROS-Based Modular End-to-End Architecture: Building and Validating a Safe and Reliable Autonomous Driving Stack

**DOI:** 10.3390/s26134269

**Published:** 2026-07-04

**Authors:** Fabio Sánchez-García, Rodrigo Gutiérrez-Moreno, Miguel Antunes-García, Santiago Montiel-Marín, Franck Fierro, Elena López-Guillén, Rafael Barea, Luis M. Bergasa

**Affiliations:** Electronics Department, University of Alcalá (UAH), 28805 Alcalá de Henares, Spain; rodrigo.gutierrez@uah.es (R.G.-M.); miguel.antunes@uah.es (M.A.-G.); santiago.montiel@uah.es (S.M.-M.); franck.fierro@uah.es (F.F.); elena.lopezg@uah.es (E.L.-G.); rafael.barea@uah.es (R.B.); luism.bergasa@uah.es (L.M.B.)

**Keywords:** autonomous driving, modular End-to-End, simulation, CARLA, holistic validation, ROS

## Abstract

The implementation of safe and reliable Autonomous Driving Stacks in complex urban environments remains a formidable engineering challenge. While classical modular pipelines provide necessary component-level interpretability, they are inherently rigid, often struggling to adapt to novel environments and failing to provide robust scene interpretation in highly interactive scenarios. In this paper, we present a modular End-to-End ROS-based autonomous driving architecture that upgrades a classical modular baseline by injecting learning-based models into its individual processing layers, integrating GaussianCaR and CLIP for dense semantic BEV perception, expanding the Hierarchical Petri Net state space for safe multi-agent reasoning, refining the planning layer with continuous curve optimization, and replacing the previous reactive controller with an Adaptive Nonlinear Model Predictive Control strategy for superior trajectory tracking. Validated in the CARLA simulator across challenging traffic scenarios and adverse environmental conditions, the proposed architecture raises the Driving Score from 53.81% to 66.46% over the previous baseline, driven by a substantial increase in the Infraction Penalty from 0.59 to 0.79, reflecting a fundamental shift towards safer and more conservative driving behavior at the cost of a moderate reduction in route completion. Against pure End-to-End approaches, our architecture achieves the highest Driving Score at 73.9% and the strongest Infraction Penalty at 0.913, demonstrating that modular interpretability and competitive End-to-End performance are not mutually exclusive. Code will be made publicly available online.

## 1. Introduction

The deployment of safe and reliable Autonomous Driving Stacks (ADS) in complex urban environments represents one of the most formidable open challenges in robotics and intelligent transportation [1]. Real-world urban driving demands that an ADS simultaneously solve tightly coupled perception, reasoning, and control problems under hard real-time constraints, in the presence of unpredictable agents, adverse weather, and safety-critical corner cases. Two fundamental barriers persist: perception pipelines must provide rich, spatially coherent scene representations dense enough to support informed decision-making, and control modules must convert this information into safe and robust driving actions, even in rare and unforeseen situations.

These requirements expose a fundamental tension between the two dominant architectural paradigms. Pure End-to-End (E2E) approaches map raw sensor inputs directly to control commands via jointly trained neural networks, benefiting from holistic optimization but sacrificing the interpretability and traceability essential for safety certification [2]. Classical modular pipelines decompose the driving task into expert-designed subsystems, enabling component-level verification, but rely on hand-crafted intermediate representations that struggle to generalize in dynamic, multi-agent scenarios. Increasingly, the field is converging on a third paradigm: Modular End-to-End architectures. Unlike pure End-to-End approaches, which map raw sensor inputs directly to control commands through a jointly optimized neural network, or classical modular pipelines, which rely entirely on hand-crafted intermediate representations between fixed subsystems, Modular End-to-End architectures inject learning-based models into key processing layers while preserving the explicit, interpretable decomposition of the driving task. In our instantiation, this manifests as data-driven perception—combining GaussianCaR, YOLOv11 and CLIP to produce dense semantic representations—whose learned outputs propagate through and inform all downstream classical modules, enabling them to operate on richer scene understanding than hand-crafted representations could provide.

In our previous work [3], we introduced a modular, ROS-based ADS achieving top-tier results among modular pipelines in the CARLA Autonomous Driving Leaderboard (CADL). Extensive holistic validation, however, revealed three critical limitations: the KDTree-based perception layer lacked the dense spatial awareness required for robust reasoning; the HIBPN decision-making layer lacked the state space to safely orchestrate complex interactive maneuvers; and the Linear Quadratic Regulator (LQR) lateral controller [4] exhibited instability when tracking sharp curves at speed, degrading both driving proficiency and passenger comfort.

**Figure 1:**
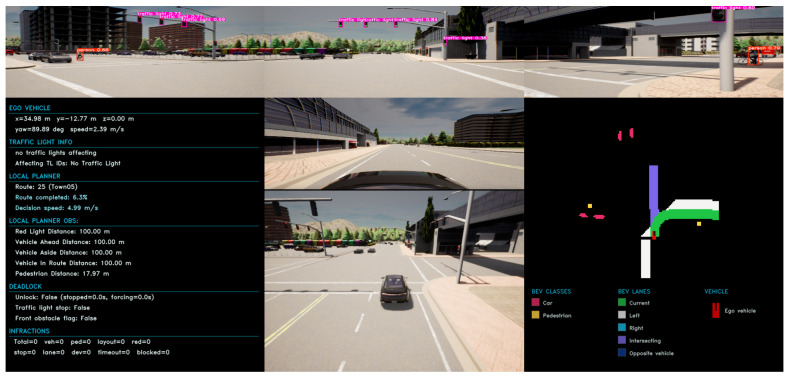
PyGame-based visualization of our Autonomous Driving stack, displaying four RGB camera streams with YOLOv11 detections and a third-person view of the ego-vehicle in the CARLA simulator. The bottom-right panel illustrates the semantic Bird’s Eye View (BEV) with lane and obstacle semantics. The bottom-left panel details the ego-vehicle state, scene understanding, and control commands.

This paper addresses each limitation directly, presenting a novel modular End-to-End ROS-based ADS that injects learning-based models into the individual processing layers without sacrificing the modularity, traceability, and Docker-based reproducibility of our established infrastructure. The result is an architecture that retains the engineering rigor required for safety-critical deployment while achieving competitive performance with pure E2E models on the CADL benchmark. Figure 1 shows the visualization of the architecture.

Building upon our previous modular ADS [3], the main contributions of this work are as follows:

- **Modular End-to-End Architecture:** A novel ROS-based ADS that preserves full component-level interpretability while injecting learning-based perception whose dense semantic representations propagate through and inform all downstream processing layers, enabling safe and traceable autonomous navigation in complex urban environments.
- **Data-Driven Perception Pipeline:** Integration of GaussianCaR [5] for dense Bird’s Eye View spatial awareness via Gaussian Splatting, coupled with the CLIP vision–language model [6] for zero-shot semantic recognition of regulatory elements, eliminating the need for domain-specific retraining.
- **Enhanced Planning and Decision-Making:** Continuous curve optimization for smooth waypoint generation, alongside an expanded HIBPN state space with new concurrent nodes for safe orchestration of overtaking, obstacle avoidance, and multi-agent lane-change maneuvers.
- **Adaptive Predictive Control:** Transition from an LQR to an Adaptive Nonlinear Model Predictive Control (NMPC) strategy [7], formulated in the Frenet frame with curvature-adaptive cost weights, significantly improving trajectory tracking accuracy, ride smoothness, and vehicle stability.

The remainder of this paper is organized as follows. Section 2 reviews the related literature across perception, decision-making, and control for autonomous driving. Section 3 describes the proposed architecture and its individual layers in detail. Section 4 presents quantitative and qualitative experimental results. Finally, Section 5 draws conclusions and outlines directions for future work.

## 2. Related Works

Developing autonomous vehicles up to SAE Level 5 remains constrained by technical, social, and regulatory challenges [8]. Because physical instrumentation is cost-prohibitive and public road testing is highly regulated, holistic validation using photorealistic simulators has become an essential paradigm. Simulators provide indispensable multi-sensor frameworks, complex traffic modeling, and automated evaluation metrics, allowing developers to test corner-case scenarios safely before real-world deployment. A complementary line of research focuses on extracting safety-critical scenarios directly from real-world crash data [9], providing empirically grounded test cases that further stress-test the robustness of autonomous driving stacks beyond standard benchmark routes. Among the available platforms, CARLA [10] has emerged as the standard for the research community, particularly due to the CADL, which provides a rigorous benchmark to evaluate agents in arbitrarily complex environments.

To tackle these challenging benchmarks, the community has traditionally adopted either pure End-to-End or classical modular software architectures. End-to-End approaches map raw sensor data directly to control commands, benefiting from joint optimization, yet lacking interpretability and traceability [11]. Notable examples of these models include CILRS [12], LBC [13], AIM [14], and NEAT [15]. However, despite their achievements in simulation, pure End-to-End pipelines currently fail to generalize and function effectively on physical vehicles in real-world environments. Conversely, classical modular pipelines divide the driving task into specialized subsystems, such as perception, planning, and control, allowing for expert specification and component-level debugging. Due to its traceability, this remains the standard approach deployed in current commercial autonomous vehicles, including Waymo [16], and industry-grade frameworks such as Autoware [17], as well as the primary modular baseline (RS) [3] evaluated in our experimental results. However, their reliance on deterministic rules and intermediate representations limits their adaptability in dynamic scenarios [18]. Consequently, to retain component-level interpretability while effectively managing the immense complexities of highly interactive environments, the adoption of modular End-to-End architectures has emerged as a highly promising paradigm [19].

Within this modular End-to-End framework, recent advancements have driven a profound shift from classical heuristics toward data-driven and predictive methods across all individual layers. In perception, the field has transitioned away from 2D/3D clustering toward unified BEV representations [20]. Innovations like 3D Gaussian Splatting [21] synthesize dense, high-fidelity spatial features, while Vision–Language Models (VLMs) such as CLIP [6] enable zero-shot semantic reasoning, drastically outperforming traditional models in recognizing regulatory elements without exhaustive retraining. Empowered by these rich contextual representations, executive layers must transcend simple reactive behaviors. While recent literature extensively explores probabilistic approaches such as Partially Observable Markov Decision Processes (POMDPs) [22] and Deep Reinforcement Learning (DRL) [23] for advanced decision-making—including highway-specific control tasks such as lane changing, ramp merging, and platoon coordination [24], as well as multi-objective eco-driving strategies that jointly optimize safety and energy efficiency [25]—we adopt HIBPNs. PNs provide a deterministic, highly interpretable, and computationally lightweight framework. This simplicity makes them ideal not only for safely orchestrating multi-agent interactions during unprotected intersections or lane changes, but also for ensuring a straightforward and safe transition to real-world physical vehicles. Finally, to execute these complex maneuvers, control strategies have evolved from classical model-driven controllers, such as PID [26], LQR [27], and standard MPC [7], towards data-driven and adaptive predictive frameworks. In this context, Adaptive MPC [28] has emerged as a robust standard. By dynamically adjusting to changing vehicle conditions and explicitly accounting for kinematics and dynamic constraints over a prediction horizon, it guarantees superior trajectory tracking and stability.

## 3. Modular End-to-End Autonomous Driving Stack

**Figure 2:**
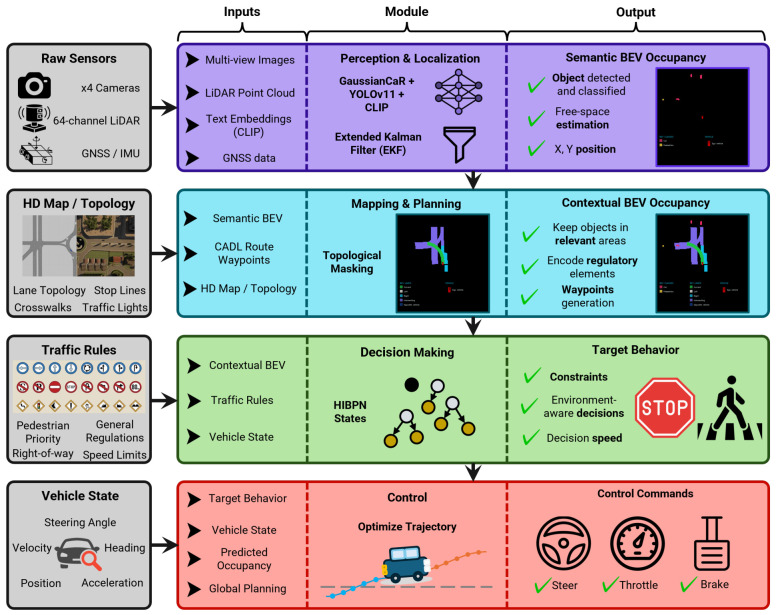
Proposed Modular End-to-End Autonomous Driving Stack. The architecture is organized into six processing layers, each receiving inputs from raw sensors or upstream modules and producing structured outputs consumed downstream. The perception and localization layers (first row) fuse multi-view camera images and LiDAR point clouds through GaussianCaR, YOLOv11, and CLIP into a semantic BEV occupancy grid, while estimating the ego-vehicle pose via GNSS, IMU, and Extended Kalman Filter (EKF) fusion. The mapping and planning layers (second row) apply topological masking to produce a contextual BEV occupancy grid and generate reference waypoints from the HD map and global route. The decision-making layer (third row) combines the contextual BEV, traffic rules, and vehicle state through the HIBPN to produce environment-aware behavioral decisions. Finally, the control layer (fourth row) optimizes the trajectory via Adaptive NMPC and outputs throttle, steer, and brake commands to the CARLA simulator.

This section describes the proposed autonomous driving architecture, evaluated within the CARLA simulator. Building upon our previous baseline [3], we preserve the modular ROS and Docker-based infrastructure while replacing classical intermediate representations with learning-based models in the key processing modules. The overall workflow is illustrated in Figure 2. The following subsections focus on the newly introduced enhancements across six layers: Localization, Planning, Mapping, Perception, Decision-Making, and Control, referring to our prior work for unaltered components.

### 3.1. Localization

Accurate and robust localization is a foundational requirement for autonomous navigation. This layer is responsible for publishing the full transform tree connecting all sensor and map frames, and for maintaining a continuous estimate of the ego-vehicle pose. Position is derived from a Global Navigation Satellite System (GNSS), providing geographic coordinates that are converted to Universal Transverse Mercator (UTM) to simplify metric calculations. Heading is supplied by an Inertial Measurement Unit (IMU). Since both sensors are subject to noise and drift, their measurements are fused through an Extended Kalman Filter (EKF) [29], which provides a stable, continuous pose estimate well-suited to the update rates required by the downstream planning and mapping layers.

### 3.2. Planning

To initiate navigation, the ADS computes the optimal global route to a destination goal. Planning is divided into two sequential components operating at different levels of abstraction: the Lane Graph Planner (LGP), responsible for topological route computation, and the Lane Waypoint Planner (LWP), responsible for generating the dense geometric reference consumed by the control layer.

#### 3.2.1. Lane Graph Planner

The LGP constructs a directed graph (DiGraph) from High-Definition (HD) map data, where nodes represent lane segments and edges encode road connectivity. Edge weights are assigned as a function of segment length and posted speed limit, reflecting both distance and expected traversal time. Given a destination goal, the globally optimal topological route is computed efficiently using Dijkstra’s algorithm, producing an ordered sequence of lane segments from the ego-vehicle’s current position to the target.

#### 3.2.2. Lane Waypoint Planner

Given the topological route produced by the LGP, the LWP generates a dense sequence of target waypoints that the control layer will track. Our previous baseline employed a simple linear discretization of lane centerlines, which introduced discontinuities in curvature and produced lateral acceleration spikes at lane transitions. To address this, we introduce a continuous curve optimization method that fits a smooth curve through the discrete lane waypoints, guaranteeing continuity of both first and second derivatives along the trajectory. The result is a geometrically consistent reference path that substantially reduces the control effort required from the downstream NMPC layer, particularly at intersections and lane merges.

### 3.3. Mapping

The mapping layer provides the ADS with structured, topologically grounded contextual awareness of its immediate environment. It comprises two submodules: the map parser and the map monitor. The map parser processes the HD map offline to extract static infrastructure relevant to navigation, including lane boundaries, intersection geometries, crosswalks, and stop lines. The map monitor then operates online, dynamically tracking the ego-vehicle’s surroundings as a function of its current pose and planned global route.

The monitored region encompasses the current and adjacent lanes, as well as complex intersection geometries including split, merge, and crossing lanes. In this work, we extend the monitor to explicitly track opposite-direction lanes, providing the contextual depth required by the decision-making layer to safely reason about oncoming traffic during overtaking and lane-change maneuvers. Additionally, the monitor extracts topological priors for all regulatory elements affecting the current route—including upcoming traffic lights, stop lines, and crosswalks—and publishes their expected 3D positions as regions of interest. This output is a critical enabler for the perception layer, allowing it to focus its recognition resources on contextually relevant signals rather than processing the full scene indiscriminately.

### 3.4. Perception

**Figure 3:**
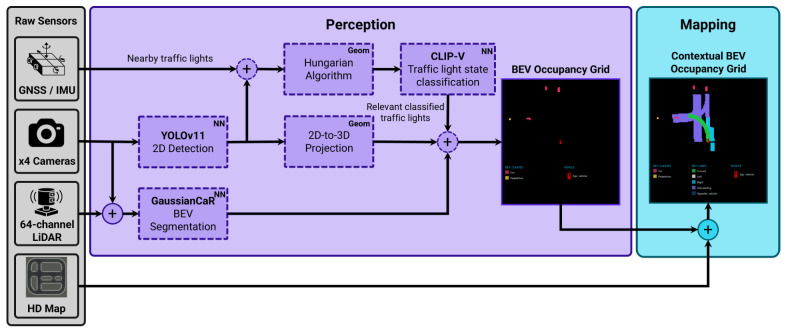
Perception pipeline combining neural (**NN**) and geometrical (**Geom**) processing stages, integrated with the mapping layer. GaussianCaR (NN) provides dense BEV segmentation from camera and LiDAR inputs. YOLOv11 (NN) detects pedestrians and traffic lights in 2D; pedestrian bounding boxes are lifted to 3D via geometric back-projection (Geom). For traffic lights, the 3D topological priors from the HD map are projected into the camera image (Geom), where the Hungarian algorithm matches each projected prior to a YOLOv11 bounding box, isolating the relevant region of interest for zero-shot state classification via CLIP (NN). All outputs are fused into a unified BEV occupancy grid, which is subsequently combined with the lane topology extracted from the HD map to produce the contextual BEV occupancy grid consumed by the decision-making layer.

The perception layer is responsible for constructing a unified, semantically rich representation of the ego-vehicle’s surroundings from raw multi-modal sensor data. It is organized around three complementary components: a dense BEV segmentation backbone (GaussianCaR), a 2D object detector (YOLOv11), and a zero-shot semantic classifier (CLIP), whose outputs are fused into a single BEV occupancy grid consumed by the decision-making layer. The full pipeline is depicted in Figure 3.

#### 3.4.1. BEV Spatial Awareness via GaussianCaR

For dense spatial awareness of surrounding traffic participants, we integrate GaussianCaR [5], a state-of-the-art End-to-End network for BEV segmentation. Its core innovation lies in repurposing 3D Gaussian Splatting as a universal view transformer, mapping raw sensor features directly into a common BEV latent space through an End-to-End differentiable pipeline, without relying on explicit depth estimation or voxelization. While originally developed for camera–radar fusion on the nuScenes dataset, we adapt it to the CADL sensor configuration by substituting the radar input with the 64-channel LiDAR available in CARLA, fine-tuning the model on camera–LiDAR data collected from the simulator to align its feature space with the CARLA sensor characteristics.

#### 3.4.2. Fine-Grained Detection via YOLOv11

While GaussianCaR provides a robust foundation for dense BEV spatial awareness, purely BEV-based models can struggle to resolve small-scale dynamic obstacles and fine-grained semantic elements, particularly pedestrians at distance. To address this, we integrate a 2D YOLOv11 detector [30] operating on the front-facing camera streams, serving a dual purpose. First, pedestrian detections are lifted from 2D image space to 3D under a ground-plane assumption: for each bounding box, the bottom-center pixel is back-projected into 3D space using the inverse intrinsic camera matrix $K^{-1}$, and the resulting position is used to populate the corresponding BEV grid cells. Second, traffic light detections are retained as 2D bounding boxes and passed downstream as candidate RoIs for regulatory element recognition.

#### 3.4.3. Zero-Shot Regulatory Element Recognition via CLIP

To determine the operational state of the traffic lights detected by YOLOv11, the expected 3D positions of the traffic lights affecting the current route—provided as topological priors by the mapping layer—are first projected into the camera image plane. The Hungarian algorithm then associates each projected prior with a candidate 2D bounding box from YOLOv11, filtering out irrelevant detections and isolating the precise RoI for each active regulatory element. Rather than training a dedicated classifier, we deploy the zero-shot semantic reasoning capabilities of the CLIP vision–language model [6]. Each isolated RoI is matched against three predefined text prompts of the form “a photo of a traffic light with a <color> light”, corresponding to red, yellow, and green states. The state yielding the highest cosine similarity in CLIP’s joint embedding space is published to the decision-making layer as the active traffic light status.

### 3.5. Decision-Making

The decision-making layer translates the continuous BEV representation produced by perception into discrete behavioral commands. This is achieved through two sequential components: an environment observer and a Hierarchical Inhibitor-Based Petri Net (HIBPN) executive core.

#### 3.5.1. Environment Observer

The environment observer bridges the gap between the continuous semantic BEV grid and the discrete state space required by the executive layer. It computes the element-wise intersection between the actor BEV grid, produced by the perception layer, and the topological lane grid, provided by the map monitor. This masking operation efficiently extracts parameterized distances and clearance flags for all surrounding actors relative to the ego-vehicle’s current and adjacent lanes, producing a compact discrete scene description that, combined with regulatory priors from mapping and perception (traffic light states, stop sign positions), fully characterizes the decision context.

#### 3.5.2. Hierarchical Petri Net Executive

The discrete scene state feeds into the HIBPN executive core. Petri Nets offer a deterministic, formally verifiable, and computationally lightweight behavioral framework, making them particularly attractive for safety-critical systems where interpretability and predictable state transitions are essential requirements. Unlike probabilistic approaches such as Partially Observable Markov Decision Processes (POMDPs) or Deep Reinforcement Learning (DRL), which optimize expected returns but offer limited formal safety guarantees, HIBPNs provide transparent, auditable decision logic that is directly transferable to physical vehicles without retraining.

Our previous baseline handled three fundamental behaviors: Lane Following, Stopping, and Intersection Yielding. Extensive validation revealed that this state space was insufficient for complex urban scenarios, leading to unsafe or suboptimal behavior in multi-agent interactions. In this work, we expand the HIBPN with three new concurrent nodes:

- **Overtaking:** Continuously monitors contiguous lanes and relative speeds to safely initiate and complete passing maneuvers around slower vehicles, verifying lane availability and clearance throughout the maneuver.
- **Obstacle Avoidance:** Triggers dynamic lateral trajectory deviations when static obstacles or Vulnerable Road Users (VRUs) partially occlude the current lane, generating a modified reference path for the control layer while continuously monitoring for clearance.
- **Lane Changing:** Evaluates global route requirements for lateral transitions to adjacent lanes, verifying the absence of actors in the target space before initiating and monitoring the maneuver to completion.

The concurrent structure of the expanded net allows multiple behavioral modes to be active simultaneously, such as executing a lane change while monitoring for oncoming traffic, without the logical deadlocks inherent in rigid finite-state machines.

### 3.6. Control

**Figure 4:**
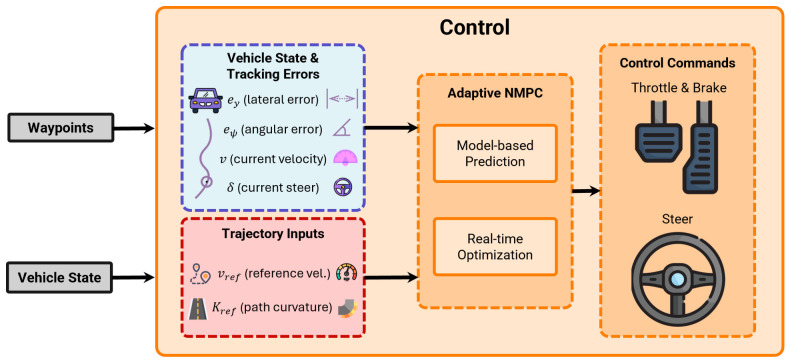
Block diagram of the proposed adaptive NMPC scheme. The tracking errors are calculated using the vehicle state and the reference trajectory, represented as waypoints. The trajectory inputs, namely the velocity and curvature references, are extracted from future waypoints. A model-based prediction and real-time optimization are used to generate throttle, steering, and brake commands.

The control layer generates the final throttle, steering, and brake commands required to accurately track the planned trajectory. Our previous LQR formulation [4] provided adequate performance on straight roads but struggled to maintain stability when tracking sharp curves at speed, resulting in jerky actuation that negatively impacted trajectory smoothness and passenger comfort. To address this, we transition to an Adaptive Nonlinear Model Predictive Control (NMPC) strategy, as illustrated in Figure 4.

#### 3.6.1. State Formulation and Frenet Frame

The controller receives the smoothed reference waypoints from the LWP and the real-time vehicle state. To formulate the optimization problem in a path-centric coordinate system that naturally decouples lateral and longitudinal dynamics, all inputs are transformed into the Frenet frame. This provides five quantities: lateral tracking error ($e_{y}$), heading error ($e_{\psi }$), current speed (*v*), steering angle (δ), reference velocity ($v_{\mathrm{ref}}$), and path curvature ($\kappa_{\mathrm{ref}}$). Based on a kinematic bicycle model, the optimizer state vector is defined as:

(1) $$x={\left[e_{y},\;e_{\psi },\;v,\;\delta \right]}^{⊤}$$

and the control input vector as:

(2) $$u={\left[a,\;\dot{\delta }\right]}^{⊤}$$

where *a* denotes longitudinal acceleration and $\dot{\delta }$ the steering rate.

#### 3.6.2. Optimization Problem

The NMPC minimizes a nonlinear least-squares cost function over a finite prediction horizon of N=40 steps with a time step of Δt=0.05s, corresponding to a two-second lookahead. Crucially, the controller is adaptive: the cost-function weights are updated online as a function of the predicted path curvature $\kappa_{\mathrm{ref}}$, tightening lateral tracking penalties in curves and relaxing them on straight segments to balance geometric precision against passenger comfort. This curvature-aware adaptation allows the controller to respond proactively to upcoming geometry rather than reactively to accumulated tracking error.

To ensure real-time feasibility, the optimization problem is solved using **acados** [7] via a Real-Time Iteration (RTI) scheme based on Sequential Quadratic Programming (SQP) with a Gauss–Newton Hessian approximation. The RTI scheme performs a single SQP iteration per control cycle, providing an efficient approximation of the optimal solution while preserving the feedback properties required for disturbance rejection.

#### 3.6.3. Command Generation

Before the prediction horizon shifts forward, the optimized first-step actions (*a* and $\dot{\delta }$) are mapped to normalized throttle, brake, and steering commands compatible with the CARLA actuator interface. Throttle and brake are derived from the signed longitudinal acceleration *a*, while the steering command is integrated from $\dot{\delta }$ and normalized to the vehicle’s steering range. This mapping ensures smooth command transitions, avoiding the actuation discontinuities that were a notable weakness of the previous LQR formulation.

## 4. Experimental Results

Experiments are conducted within the CARLA Autonomous Driving Leaderboard (CADL), which evaluates an ADS across arbitrarily complex environments including urban districts, highways, residential areas, and unsigned intersections, under varying traffic densities and weather conditions. All local tests were executed on a high-performance desktop PC (Intel Core i9-14900K, 32 GB RAM, NVIDIA GeForce RTX 4090 24 GB VRAM) running ROS Noetic.

### 4.1. Metrics

Following the standard CADL evaluation protocol, three metrics are used to holistically assess the performance of the autonomous driving agent:

1. **Route Completion (RC):** measures the percentage of route distance successfully completed by the agent, averaged across *N* routes. Driving outside lane boundaries is penalized through a multiplicative factor proportional to the fraction of distance covered off-road, ensuring that out-of-bounds navigation does not artificially inflate the score.
  (3) $$RC=\frac{1}{N}\sum_{i=1}^{N}R_{i}$$
  where *N* is the number of routes and $R_{i}\in \left[0,1\right]$ denotes the route completion score for the *i*-th route.
2. **Infraction Penalty (IP):** aggregates all infractions triggered by the agent into a single safety score in [0,1], where 1.0 indicates a fully infraction-free run. Each infraction type *j* contributes proportionally to its severity through a predefined penalty coefficient $c_{j}$, with values set to 0.50 for pedestrian collisions, 0.60 for vehicle collisions, 0.65 for static object collisions, 0.70 for red light violations, and 0.80 for stop sign violations.
  (4) $$IP=\frac{1}{1+\sum_{j}c_{j}\cdot n_{j}}$$
  where *j* indexes the infraction type, $c_{j}$ is the corresponding penalty coefficient, and $n_{j}$ is the number of infractions of type *j* committed by the agent.
3. **Driving Score (DS):** the primary leaderboard metric, computed as the mean product of RC and IP across all routes. It jointly rewards route completion and safe behavior, with a maximum value of 100.
  (5) $$DS=\frac{1}{N}\sum_{i=1}^{N}R_{i}P_{i}$$
  where $P_{i}\in \left[0,1\right]$ is the infraction penalty score for the *i*-th route.

### 4.2. Agent Setup

**Figure 5:**
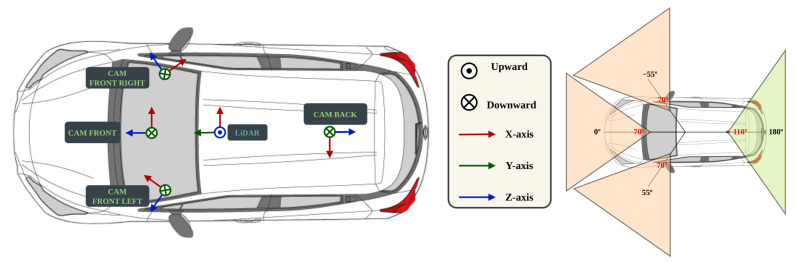
Sensor layout of the ego-vehicle, comprising four monocular RGB cameras (three forward-facing at 70° FoV and one rear-facing at 110° FoV), a 64-channel LiDAR, a GNSS, a speedometer, and an IMU. **Left**: top-down and side placement diagram. **Right**: camera field of view coverage.

Our sensor configuration comprises four monocular RGB cameras (three forward-facing to cover a wide panoramic field of view, and one rear-facing), a 64-channel LiDAR, a GNSS, a speedometer, and an IMU. All cameras operate at a resolution of 1600×900 pixels; the three front-facing cameras share a field of view of 70°, while the rear-facing camera uses a wider 110° field of view, mimicking the camera configuration of the nuScenes dataset [31]. The sensor placement and disposition are illustrated in Figure 5.

### 4.3. Experiments

Our evaluation is divided into two main experiments. Experiment A demonstrates the internal evolution of our ADS by comparing our classical modular baseline [3] against the proposed modular End-to-End architecture. Experiment B contextualizes our results against pure state-of-the-art End-to-End approaches. A deeper analysis of the compared baseline architectures can be found in our previous work [3].

In Experiment A, both architectures are evaluated across five routes in six CARLA towns featuring roundabouts, tunnels, and complex junctions. Table 1 reports the results of the previous baseline, which relied on KDTree LiDAR clustering and LQR control. Despite an acceptable global RC of 93.28%, the architecture struggled in dense and interactive scenarios, reflected in a low IP of 0.59 and a global DS of 53.81%. Table 2 reports the results of the proposed architecture. The integration of dense BEV perception, Adaptive NMPC control, and expanded HIBPNs collectively raises the DS to 66.46%, representing a significant improvement over the baseline. This gain is driven primarily by a substantial increase in IP from 0.59 to 0.79, reflecting a meaningfully safer driving behavior: the system detects and responds to more hazards, stops more conservatively, and commits far fewer infractions. While this comes at the cost of a reduction in RC from 93.28% to 85.54%, the overall result is unambiguous—the proposed architecture is considerably safer, and the improvement in driving safety more than compensates for the reduction in route completion, as evidenced by the higher DS.

In Experiment B, our architecture is compared against four pure End-to-End models—CILRS, LBC, AIM, and NEAT—on a benchmark of 42 routes under varied weather conditions, following the protocol of [15]. Results are reported as mean and standard deviation over three independent evaluations in Table 3, which additionally reports the sensor configuration of each method. CILRS and LBC rely solely on monocular camera input, while AIM and NEAT augment cameras with GPS and IMU; our architecture further incorporates a 64-channel LiDAR, the primary sensor advantage to consider when interpreting the results. Our approach achieves the highest DS at 73.9%, driven by the strongest IP among all evaluated methods at 0.913. As observed in Experiment A, the lower RC reflects the more conservative nature of our agent: an agent that stops in uncertain situations and respects regulatory elements inherently completes fewer route segments. The higher DS confirms this trade-off is favorable, demonstrating that within a modular End-to-End framework, improved safety and competitive task performance are not mutually exclusive.

### 4.4. Ablation Study

#### 4.4.1. Holistic Driving Metrics

To isolate the contribution of each individual upgrade introduced in this work, we conduct an incremental ablation study starting from the classical modular baseline [3] and progressively incorporating each new module. Rather than evaluating all possible combinations, we follow a sequential integration strategy that mirrors the architectural dependency order of the stack: planning generates the reference path consumed by the control layer, while perception produces the semantic BEV representation consumed by the decision-making layer. Evaluating combinations that bypass this dependency order would conflate the contributions of individual modules and produce results that are architecturally inconsistent; the chosen sequential strategy therefore provides the most interpretable isolation of each module’s contribution within the constraints of the pipeline. Each configuration is evaluated under the same conditions and routes as Experiment A, and results are reported in terms of DS, RC, and IP to provide a holistic view of the impact of each upgrade.

The addition of the upgraded planning layer alone yields improvements in both RC and IP, as shown in Table 4. The smoother waypoint generation provides a geometrically cleaner reference path that the agent can follow more reliably, reducing minor trajectory deviations without altering the overall driving behavior.

Incorporating the perception upgrade further increases the IP, driven primarily by the improved traffic light detection and classification pipeline, which successfully identifies traffic lights that the previous architecture missed, as well as by the richer spatial awareness of surrounding vehicles in adjacent lanes. This improvement is accompanied by a moderate reduction in RC, attributable to occasional false detections or minor misclassifications that cause the agent to stop unnecessarily. Despite this, a cumulative DS improvement of 8.73% over the baseline confirms that the perceptual gains outweigh these limitations.

Finally, the addition of the expanded decision-making module produces a similar pattern: the more conservative behavioral logic of the HIBPNs introduces additional stops in uncertain situations, further reducing RC, while simultaneously driving a significant increase in IP as the agent commits fewer infractions in complex interactive scenarios. Taken together, these results confirm that each module contributes meaningfully to the overall improvement in driving safety, and that the progressive shift towards more conservative and infraction-aware behavior across the stack is the key factor behind the superior DS achieved by the full architecture.

#### 4.4.2. Comfort and Tracking Metrics

To evaluate the contribution of the control layer upgrade, both the LQR and Adaptive NMPC controllers were assessed in CARLA on an urban route of approximately 500 m at a maximum speed of 50 km/h, with control signals updated at 10 Hz. To obtain representative comfort-related metrics, the recorded signals were smoothed using a low-pass filter to attenuate high-frequency oscillations. Lateral tracking, steering smoothness, and passenger comfort metrics were computed along the complete route and are reported in Table 5.

The Adaptive NMPC controller outperforms the LQR baseline across all key metrics, reducing maximum lateral error by 60% and lateral jerk by approximately 72%. Steering oscillations are reduced by half, indicating significantly smoother lateral control actions. Although the LQR achieves marginally lower longitudinal jerk, both controllers remain within comfortable driving limits for urban scenarios.

### 4.5. Qualitative Results

To complement the quantitative evaluation, we present a series of qualitative results across representative scenarios, illustrating both the overall behavior of the proposed architecture and its improvements over the previous baseline.

#### 4.5.1. Overall Behavior

**Figure 6:**
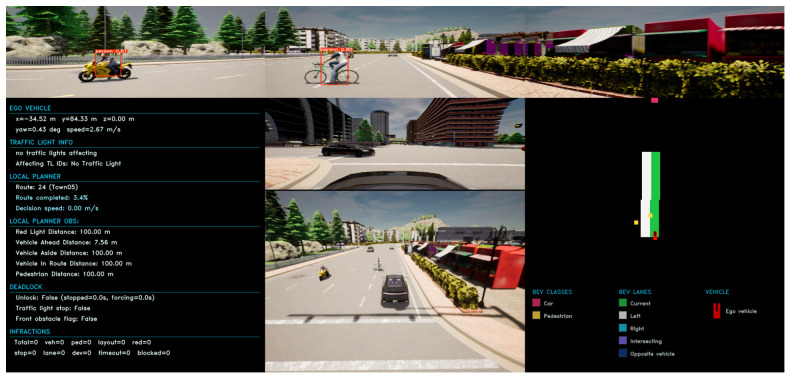
Visualization output during an emergency stop scenario triggered by a cyclist unexpectedly crossing the lane. The agent successfully detects the vulnerable road user and reflects the ego-vehicle’s stopped state in both the local planner observations and the BEV representation, which accurately segments the drivable lanes and surrounding lane semantics.

Figure 6 illustrates the system’s response in a safety-critical scenario: upon detecting a VRU suddenly crossing the drivable area, the architecture successfully triggers an emergency stop maneuver, demonstrating the spatial awareness provided by GaussianCaR.

**Figure 7:**
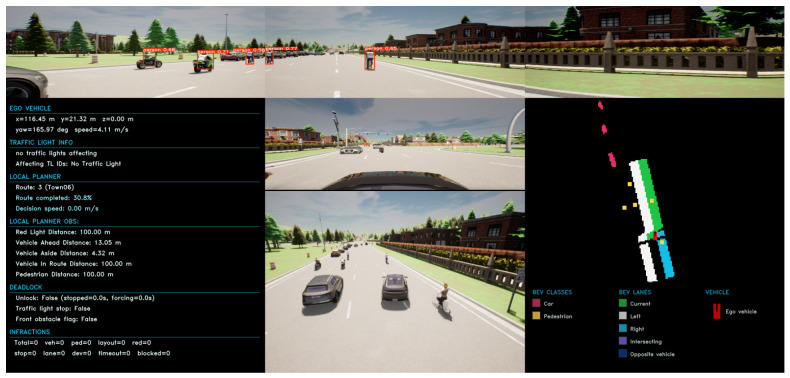
Visualization output during a dynamic lane change maneuver. The ego-vehicle actively monitors the adjacent lane, correctly identifies a gap in traffic, and safely completes the transition while yielding to an approaching vehicle. The BEV representation on the right reflects the surrounding lane semantics and the detected vehicle in the target lane.

Figure 7 presents a representative lane change maneuver in which the expanded HIBPN state space enables the ego-vehicle to assess the adjacent lane, identify a safe gap, and complete the transition without incident.

#### 4.5.2. Planning Improvement

**Figure 8:**
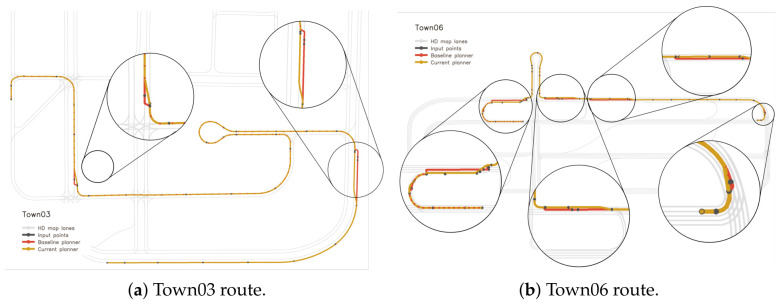
Comparison of the baseline planner (red) and the proposed planner (orange) on two different routes. Zoomed insets highlight the improved smoothness at lane transitions and sharp curves.

As demonstrated in the ablation study, one of the primary benefits of the upgraded planning layer is the smoothing of lane transitions and curve waypoints. Figure 8 illustrates this improvement on representative routes of Town03 and Town06, where the difference between the baseline planner and the proposed planner is most visible at sharp transitions.

#### 4.5.3. Traffic Light Recognition

**Figure 9:**
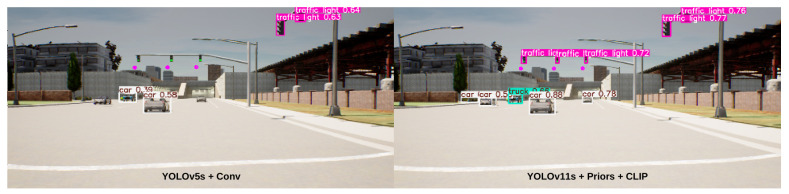
Comparison of traffic light recognition in a challenging long-range scenario, where signals are located at distances exceeding 20 m from the ego-vehicle. Magenta dots denote the topological priors extracted by the mapping layer. **Left**: the previous pipeline (YOLOv5s with a convolutional classifier) misses several distant signals and produces low-confidence detections. **Right**: the proposed pipeline (YOLOv11 with Hungarian-based prior association and CLIP zero-shot classification) correctly detects and classifies all active traffic lights within the scene.

North American traffic light topologies present a particularly challenging scenario for perception systems due to the large distances at which signals are typically positioned. Figure 9 illustrates a representative case where the proposed pipeline demonstrates clear improvements in detection reliability and classification accuracy over the previous architecture.

#### 4.5.4. Failure Case Analysis

Despite the overall improvements demonstrated above, the proposed architecture exhibits identifiable failure modes that account for the observed reduction in RC. The primary cause is occasional misclassification by the CLIP-based traffic light classifier at long range: when traffic lights are distant, the isolated RoI crops are of insufficient resolution for reliable zero-shot classification, leading the agent to interpret ambiguous signals conservatively and halt unnecessarily. A secondary and less frequent failure mode involves false positive detections at the borders of the drivable area, where the perception pipeline occasionally segments non-existent obstacles, causing the agent to perform unnecessary stops. Both failure modes reflect the inherent limitations of zero-shot and purely appearance-based perception approaches.

## 5. Conclusions and Future Works

This paper presents a modular End-to-End autonomous driving architecture that upgrades a classical modular baseline [3] by injecting learning-based models into its individual processing layers. The proposed system combines GaussianCaR and CLIP for dense semantic BEV perception, an expanded HIBPN state space for safe multi-agent reasoning, continuous curve optimization for smoother waypoint generation, and an Adaptive NMPC controller for precise trajectory tracking, all within the original modular ROS and Docker-based infrastructure.

Validation in the CARLA simulator demonstrates that the architecture significantly outperforms the previous baseline, raising the Driving Score from 53.81% to 66.46% and the Infraction Penalty from 0.59 to 0.79. A key finding of this work is that these gains come hand in hand with a fundamental shift in driving behavior: rather than attempting every maneuver regardless of risk, the proposed architecture is explicitly designed to prioritize safety through its offline architectural choices—conservative HIBPN behavioral logic, perception-driven hazard detection, and curvature-aware NMPC formulation—stopping in uncertain situations and respecting regulatory elements that the previous system would have ignored. The resulting reduction in Route Completion is the observable signature of this shift, and the higher Driving Score confirms it is a worthwhile trade-off. This also confirms that minimizing infractions is a more valuable objective than maximizing route progress, and that safety and task performance are not mutually exclusive within a modular End-to-End framework. Against pure End-to-End approaches, our architecture achieves the highest Driving Score at 73.9% and the strongest Infraction Penalty at 0.913, further validating this principle.

Future work will focus on bridging the sim-to-real gap through physical deployment on a real instrumented vehicle, optimizing the BEV perception pipeline for embedded hardware, and integrating predictive trajectory modeling for surrounding agents into the decision-making layer to further reduce infractions in complex interactive scenarios.

## Figures and Tables

**Table 1:** Local Experiment A.1: Previous Baseline Architecture (KDTree Perception, LQR Control).

Town	RL [m]	DS ↑ [%]	RC ↑ [%]	IP ↑ [0, 1]
Town01	680.04	86.00	100.00	0.86
Town02	899.57	45.17	88.09	0.54
Town03	1520.76	56.44	86.56	0.70
Town04	2066.07	52.60	98.27	0.53
Town05	1043.06	38.42	86.76	0.46
Town06	1655.34	44.24	100.00	0.44
**Global**	**1310.81**	**53.81**	**93.28**	**0.59**

**Table 2:** Local Experiment A.2: Proposed Modular End-to-End Architecture (GaussianCaR, CLIP, Adaptive NMPC, Opposite-Lane Monitoring). Best results per column are shown in **bold**. ↑ indicates that higher values correspond to better performance.

Town	RL [m]	DS ↑ [%]	RC ↑ [%]	IP ↑ [0, 1]
Town01	680.04	88.00	100.00	0.88
Town02	899.57	52.40	72.30	0.73
Town03	1520.76	68.50	90.10	0.76
Town04	2066.07	63.20	84.50	0.75
Town05	1043.06	57.80	76.40	0.76
Town06	1655.34	68.86	89.94	0.86
**Global**	**1310.81**	**66.46**	**85.54**	**0.79**

**Table 3:** Local Experiment B: Comparison against state-of-the-art End-to-End architectures (μ and σ over 3 evaluations). *Aux. Sup.* denotes the Auxiliary Supervision signal used during training. *Cam.* denotes the number of RGB cameras. Best results per column are shown in **bold**. ↑ indicates that higher values correspond to better performance.

Method	Aux. Sup.	Cameras	LiDAR	DS ↑ [%]	RC ↑ [%]	IP ↑ [0, 1]
CILRS [12]	Velocity	1	✗	22.97 ± 0.90	35.46 ± 0.41	0.66 ± 0.02
LBC [13]	BEV Sem	1	✗	29.07 ± 0.67	61.35 ± 2.26	0.57 ± 0.02
AIM [14]	None	3	✗	51.25 ± 0.17	70.04 ± 2.31	0.73 ± 0.03
	2D Sem			57.95 ± 2.76	80.21 ± 3.55	0.74 ± 0.02
AIM-MT	BEV Sem	3	✗	60.62 ± 2.33	77.93 ± 3.06	0.78 ± 0.01
	Dth+2D Sem			64.86 ± 2.52	80.81 ± 2.47	0.80 ± 0.01
NEAT [15]	BEV Sem	3	✗	65.10 ± 1.75	79.17 ± 3.25	0.82 ± 0.01
RS [3]	Modular	4	✗	62.91 ± 1.96	**92.11 ± 1.84**	0.69 ± 0.01
**Ours**	Modular E2E	4	✓	**73.9 ± 0.83**	78.6 ± 1.76	**0.913 ± 0.02**

**Table 4:** Ablation study showing the incremental contribution of each upgraded module over the classical modular baseline. ΔDS denotes the cumulative improvement in Driving Score with respect to the baseline. Best results per column are shown in **bold**.

Planning	Perception	Decision Making	DS ↑ [%]	ΔDS ↑ [%]	RC ↑ [%]	IP ↑ [0, 1]
			53.81	—	93.28	0.59
✓			59.41	+5.61	**94.23**	0.64
✓	✓		62.54	+8.73	91.06	0.70
✓	✓	✓	**66.46**	+12.69	85.54	**0.79**

**Table 5:** Comparison between LQR and NMPC controllers. ↓ indicates that lower values correspond to better performance.

Metric	LQR	NMPC
Maximum Lateral Error ↓ [m]	0.025	0.010
Steering Oscillation RMS ↓ [deg]	0.010	0.005
Lateral Jerk RMS Steering ↓ [m/s^3^]	0.057	0.016
Longitudinal Jerk RMS Filtered ↓ [m/s^3^]	0.022	0.034
Control Effort RMS ↓ [-]	0.424	0.393

## Data Availability

The original contributions presented in this study are included in the article. Further inquiries can be directed to the corresponding author.
